# Serine–glycine-one-carbon metabolism: vulnerabilities in MYCN-amplified neuroblastoma

**DOI:** 10.1038/s41389-020-0200-9

**Published:** 2020-02-07

**Authors:** Erhu Zhao, Jianbing Hou, Hongjuan Cui

**Affiliations:** 1 State Key Laboratory of Silkworm Genome Biology, College of Biotechnology, Southwest University, Chongqing, 400716 China; 2 Cancer Center, Medical Research Institute, Southwest University, Chongqing, 400716 China; 3 Engineering Research Center for Cancer Biomedical and Translational Medicine, Southwest University, Chongqing, 400716 China; 4 Chongqing Engineering and Technology Research Center for Silk Biomaterials and Regenerative Medicine, 400715 Chongqing, China

**Keywords:** Cancer metabolism, Cell signalling

## Abstract

In a recent study published in *Cancer Research*, Xia and colleagues reported that, in cancer, constituents in serine–glycine-one-carbon (SGOC) metabolism exhibit enhanced transcriptional activation and are increasingly utilised, which results in more glucose-derived carbon to serine–glycine biosynthesis. The current work identifies an MYCN-dependent metabolic vulnerability and shows a variety of associations between metabolic reprogramming and enhanced sensitivity to metabolic stress, which may lead the way to unlocking new anticancer therapies. Here, we summarised new insights into the role of SGOC metabolism in the progression of neuroblastoma (NB) with highly activated SGOC metabolism.

## Introduction

Neuroblastoma (NB) is a highly heterogeneous disease in children that ranges from low-risk tumours that spontaneously differentiate and regress with minimal or no therapy to aggressive high-risk tumours that have a poor prognosis, frequently metastasise and are resistant to therapy. Child survivors often suffer a lifetime of severe adverse effects elicited by chemotherapeutic drug toxicity. Xia and colleagues reported a MYCN-dependent metabolic vulnerability that could suppress the serine–glycine-one-carbon (SGOC) pathway, which offers potential targets for preventing therapeutic resistance in NB. This mechanism may result in fewer required drugs for treatment, thereby reducing the incidence of deleterious adverse effects in the paediatric patients.

*MYCN* is an important member of the nuclear proto-oncogene *MYC* family, which supports the development of many different tumours, including retinoblastoma, medulloblastoma, prostate cancer, lung cancer and NB. The *MYCN* oncogene is frequently amplified in malignant NB, and this amplification is considered the single most relevant genetic alteration that promotes cell proliferation, metabolic changes and aggressiveness in NB. Many malignancies would benefit from improved MYCN-targeting therapeutic approaches. Several indirect strategies to target MYCN-driven tumorigenesis exist as follows: (a) *MYCN* transcription targeting; (b) MYCN protein stability targeting; (c) MYCN dimerisation and transcriptional activity targeting; (d) MYCN-based immunotherapy; and (e) indirect MYCN targeting^1^. Xia and colleagues^2^ uncovered a mechanism of aberrant SGOC pathway activation in NB with MYCN amplification, and their findings may provide potential therapeutic targets using three of the abovementioned strategies (excluding immunotherapy).

## The Serine–glycine-one-carbon metabolic unit

The one-carbon metabolic pathway can generate various molecules, e.g., nucleotides, proteins, and lipids and substrates for methylation reactions, as well as maintain the redox status; this process comprises three critical reactions: the folate cycle, the methionine cycle and the trans-sulphuration pathway. Serine and glycine are synthesised de novo from glycolysis through oxidation of the intermediate 3-PGA, and these products fuel one-carbon metabolism.

The SGOC metabolic unit, which includes serine, glycine and one-carbon metabolism, supplies an integration point in cellular metabolism that permits cells to achieve diverse biological functions by converting serine and glycine into several other metabolic products. Locasale considered that serine, glycine and the one-carbon cycle form a vital full circle in cancer metabolism^3^. Mehrmohamadi and colleagues first proposed the concept of the SGOC metabolic network^4^ and identified the expansive and heterogeneous functions of this network in human cancer. They showed the first comprehensive systems-level analysis of the expression pattern, metabolic flux, and the correlation with SGOC metabolism and delineated the potential features of the SGOC pathway in ovarian, lung, colorectal, and breast cancer. Unlike the glycolytic network which may be universally overexpressed in the whole network of cancers, the expressions of SGOC-network constituents differ in more complicated ways. They found that SGOC heterogeneity is strikingly apparent, which may confer its predisposed activation in cancer.

**Fig. 1:**
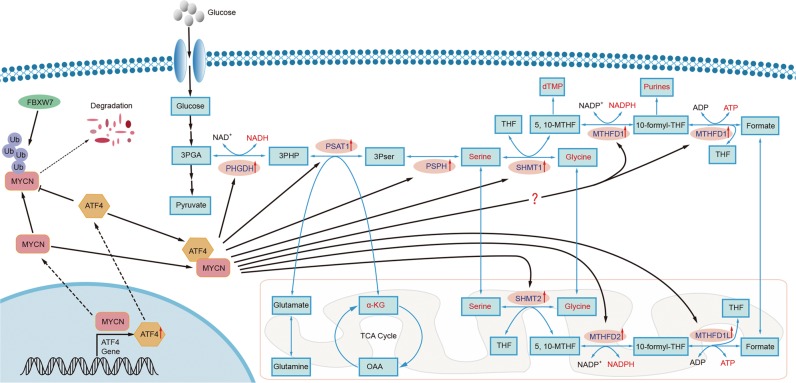
The regulatory mechanisms for the activation of serine–glycine-one-carbon metabolism in MYCN-amplified neuroblastoma. Blue text indicates SGOC enzymes; red text indicates the key products of the SGOC pathway; the rounded rectangle indicates the mitochondrion; and the fan shape indicates a part of the cell nucleus.

In their previous work, Ding^5^, Zhao^6^ and Liu^7^ showed that serine–glycine metabolism is essential for tumorigenesis. Importantly, they confirmed that ATF4 is a vital regulator of the transcriptional activation of serine–glycine metabolism and the stimulation of cell proliferation. Ding and Zhao identified a genetic mechanism for activating the serine–glycine biosynthetic pathway. Liu and colleagues used the TH-MYCN mouse, a transgenic model of high-risk NB with MYCN overexpression, to show that the serine–glycine synthesis pathways are essential for tumorigenesis. Importantly, Xia and colleagues reported the potential roles of the SGOC pathway based on their previous research in MYCN-amplified NB; these roles may be the hidden Achilles heel of all MYCN-driven cancers. In this report, Xia and colleagues found that SGOC pathway activation requires both MYCN and ATF4 in MYCN-amplified NB, and these factors form a positive feedback loop. The MYCN protein can activate the expression of ATF4. In return, ATF4 can enhance the stability of MCYN by inhibiting the MYCN ubiquitination mediated by FBXW7 (Fig. 1).

SGOC metabolism incorporates serine–glycine biosynthesis, one-carbon metabolism, and purine nucleotide biosynthesis in a positive feedback loop. The enzymes in the SGOC pathway include PHGDH, PSAT1, PHSH, SHMT1, SHMT2, MTHFD1, MTHFD1L and MTHFD2, all of which associate the glycolysis intermediate metabolite 3PGA with serine–glycine biosynthesis and two carriers of one carbon: 5,10-MTHF and 10-formyl-THF. In purine nucleotide biosynthesis, the carbons are donated not only by glycine, but also by 10-formyl-THF; while the nitrogen moieties are contributed only by glycine. Furthermore, 5,10-MTHF is also a prerequisite for the de novo thymidylate production, which is an important coenzyme of thymidylate synthase. A growing body of evidence has indicated that SGOC pathway activation is a considerable part of cancer metabolism. Xia and colleagues confirmed that serine–glycine biosynthesis, one-carbon metabolism, and purine nucleotide biosynthesis, which are linked by the SGOC pathway, were the most markedly increased metabolic processes in MYCN-amplified NB. Furthermore, they found that higher levels of SGOC gene expression are significantly linked to tumour stage advancement and unfavourable prognosis in MYCN-amplified NB patients.

## Control of serine–glycine-one-carbon metabolism in MYCN-amplified neuroblastoma

All members of the proto-oncogene MYC family can bind to the DNA core sequence CANNTG. Xia and colleagues found that enzyme genes within the SGOC pathway, such as PHGDH and PSAT1, contain the short conserved sequence CACGTG within either their promoters or first introns. They also used ChIP-qPCR and other methods to identify the SGOC enzyme genes as direct transcriptional targets of MYCN. Moreover, ATF4 plays a vital role on the transcriptional activation of metabolic enzymes in the serine–glycine biosynthesis pathway. Xia and colleagues confirmed that ATF4 plays a fundamental role in sustaining SGOC enzyme gene expression and maintaining tumour cell growth and proliferation. Importantly, they found that the SGOC enzyme genes are also direct transcriptional targets of ATF4. More specifically, in MYCN-amplified NB, both MYCN and ATF4 are the essential for transcriptional activation of SGOC enzyme genes. Xia and colleagues used ChIP-qPCR and other methods to show that ATF4 is the downstream target of MYCN in the transcriptional control of SGOC enzyme genes. Furthermore, they found that ATF4 overexpression antagonised the effect of FBXW7, resulting in up-regulated MYCN expression and inhibited MYCN ubiquitination. In contrast, FBXW7 silencing had no influence on the expression of MYCN mRNA but markedly enhanced MYCN protein expression, resulting in higher PHGDH expression. Moreover, when FBXW7 expression was silenced, ATF4 downregulation could no longer enhance the ubiquitination of MYCN. Finally, they demonstrate that a MYCN-ATF4 feedback loop modulates the transcriptional activity of the SGOC enzyme gene in NB cells with MYCN amplification.

PHGDH is the first rate-limiting key metabolic enzyme in the serine–glycine biosynthesis pathway and has been linked to tumorigenesis; the outputs include sphingolipid, nucleotide, glutathione biosynthesis and the folate pathway (one-carbon metabolism)^8^. Xia and colleagues found that the SGOC genes are transcriptionally activated in NB cells with MYCN amplification but not those without MYCN amplification. Xia and colleagues also used two small molecule inhibitors of PHGDH, NCT-503^9^ and CBR-5884^10^ and confirmed that MYCN sensitises NB cells to PHGDH inhibition.

Furthermore, the SGOC pathway connects glycolytic reactions with one-carbon metabolism, the biosynthesis of serine and glycine. Xia and colleagues used a ^13^C mass-isotopomer distribution model to show that NB cells with MCYN amplification utilised more glucose-derived carbon for serine and glycine biosynthesis than those without, which suggests a metabolic basis for their differential sensitivities to PHGDH inhibition. They further found that PHGDH inhibition induces metabolic stress and leads to G1 arrest and autophagy in NB cells with MYCN amplification but not those without MYCN amplification. Because PHGDH is the first rate-limiting key metabolic enzyme in the SGOC pathway, PHGDH inhibition may create a metabolic vulnerability in tumours with a highly activated SGOC metabolism. These findings support a recent publication from Reina-Campos and colleagues revealing that PHGDH inhibition is a vulnerability in neuroendocrine prostate cancer that can be exploited therapeutically by targeting the SGOC pathway^11^.

## Conclusions

Taken together, these findings by Xia and colleagues reveal new insight into the features of SGOC metabolism in the progression of NB with MYCN amplification or cancers with highly activated SGOC metabolism. The SGOC metabolic network is important not only for nucleotide, protein, and lipid biosynthesis but also for DNA methylation, histone methylation and redox balance. The outputs of SGOC metabolism are required for cell viability and proliferation and are particularly likely to be co-opted by aggressive cancers. Targeting SGOC metabolism may be an effective and less toxic approach to improve outcomes in MYCN-amplified NB patients. The enzymes within the SGOC pathway may be powerful prognostic tools and perhaps clinically relevant targets in all highly SGOC-activated tumours in the future.
